# Performance of a Biodegradable Composite with Hydroxyapatite as a Scaffold in Pulp Tissue Repair

**DOI:** 10.3390/polym12040937

**Published:** 2020-04-17

**Authors:** Motoki Okamoto, Sayako Matsumoto, Ayato Sugiyama, Kei Kanie, Masakatsu Watanabe, Hailing Huang, Manahil Ali, Yuki Ito, Jiro Miura, Yujiro Hirose, Koichiro Uto, Mitsuhiro Ebara, Ryuji Kato, Aika Yamawaki-Ogata, Yuji Narita, Shigetada Kawabata, Yusuke Takahashi, Mikako Hayashi

**Affiliations:** 1 Department of Restorative Dentistry and Endodontology, Osaka University Graduate School of Dentistry, 1-8, Yamadaoka, Suita, Osaka 565-0871, Japan; m.sayako@dent.osaka-u.ac.jp (S.M.); masa_watanabe@dent.osaka-u.ac.jp (M.W.); helenstory93@dent.osaka-u.ac.jp (H.H.); manahilali@dent.osaka-u.ac.jp (M.A.); yuki-ito@dent.osaka-u.ac.jp (Y.I.); takahasi@dent.osaka-u.ac.jp (Y.T.); mikarin@dent.osaka-u.ac.jp (M.H.); 2 Department of Basic Medicinal Science, Nagoya University, Chikusa, Nagoya 464-8601, Japan; sugiyama.ayato@k.mbox.nagoya-u.ac.jp (A.S.); kanie-k@ps.nagoya-u.ac.jp (K.K.); kato-r@ps.nagoya-u.ac.jp (R.K.); 3 Division for Interdisciplinary Dentistry, Osaka University Dental Hospital, 1-8 Yamadaoka, Suita, Osaka 565-0871, Japan; miura_j@dent.osaka-u.ac.jp; 4 Department of Oral and Molecular Microbiology, Osaka University Graduate School of Dentistry, Suita, Osaka 565-0871, Japan; yujirohirose@dent.osaka-u.ac.jp (Y.H.); kawabata@dent.osaka-u.ac.jp (S.K.); 5 International Center for Materials Nanoarchitectonics (WPI-MANA), National Institute for Materials Science (NIMS), 1 Chome-1 Namiki, Tsukuba, Ibaraki 305-0044, Japan; UTO.Koichiro@nims.go.jp (K.U.); EBARA.Mitsuhiro@nims.go.jp (M.E.); 6 Institute of Nano-Life-Systems, Nagoya University, Furo-cho, Chikusa-ku, Nagoya 464-8603, Japan; 7 Department of Cardiac Surgery, Nagoya University Graduate School of Medicine, 65 Tsurumai-cho, Showa-ku, Nagoya 466-8550, Japan; aika@med.nagoya-u.ac.jp (A.Y.-O.); ynarita@med.nagoya-u.ac.jp (Y.N.)

**Keywords:** pulp capping, tertiary dentin, polymer complex, RNA sequence

## Abstract

Vital pulp therapy is an important endodontic treatment. Strategies using growth factors and biological molecules are effective in developing pulp capping materials based on wound healing by the dentin-pulp complex. Our group developed biodegradable viscoelastic polymer materials for tissue-engineered medical devices. The polymer contents help overcome the poor fracture toughness of hydroxyapatite (HAp)-facilitated osteogenic differentiation of pulp cells. However, the composition of this novel polymer remained unclear. This study evaluated a novel polymer composite, P(CL-*co*-DLLA) and HAp, as a direct pulp capping carrier for biological molecules. The biocompatibility of the novel polymer composite was evaluated by determining the cytotoxicity and proliferation of human dental stem cells in vitro. The novel polymer composite with BMP-2, which reportedly induced tertiary dentin, was tested as a direct pulp capping material in a rat model. Cytotoxicity and proliferation assays revealed that the biocompatibility of the novel polymer composite was similar to that of the control. The novel polymer composite with BMP-2-induced tertiary dentin, similar to hydraulic calcium-silicate cement, in the direct pulp capping model. The BMP-2 composite upregulated wound healing-related gene expression compared to the novel polymer composite alone. Therefore, we suggest that novel polymer composites could be effective carriers for pulp capping.

## 1. Introduction

Dental pulp tissue is surrounded by dentin, the soft connective tissue that provides nutrition and controls biological reactions, including defense against bacteria. It responds to various stimuli and undergoes a repair process, inducing hard tissue formation [1]. Pulp exposed by caries or trauma needs to be protected with appropriate pulp capping material to maintain its vitality and function, which is called vital pulp therapy. Preserving pulp vitality is one of the important endodontic treatments and has attracted attention in recent years [2]. The importance of preserving pulp is predictable as material developments evolve. An ideal pulp capping material should isolate the pulp from infection and provide an ideal biological environment for dental pulp tissue repair [3]. ProRoot mineral trioxide aggregate (MTA; Dentsply Sirona, Ballaigues, Switzerland), a hydraulic calcium-silicate cement, has been widely accepted as the gold standard for direct pulp capping in dental clinical practice [4]. However, concerns regarding MTA, including mixture inconsistency, tooth discoloration, lack of adhesion to dentine, prolonged setting time, and especially handling inconvenience, limit its application in dental clinical practice [5]. Additionally, the mechanism of MTA in the pulpal wound healing process is not completely undissolved. Furthermore, the underlying mechanisms of pulp tissue healing also are still unclear, and critical targets for the development of evidence-based direct pulp capping materials need to be identified.

Direct pulp capping strategies using growth factors and biological molecules are effective [6,7]. A combination of several growth factor carriers has been shown to be effective at inducing pulp hard tissue [8]. Regarding the carrier, most previous studies report using gelatin sponge or gel; however, long-term prognosis may be uncertain owing to problems in operability and mechanical strength [9], as direct capping materials need a certain amount of strength for long-term maintenance and stability [10].

Hydroxyapatite (HAp) is a component of natural bone and teeth, and has long been studied as a scaffold for pulp capping. HAp is considered useful for pulp capping owing to its osteoinductive properties, high mechanical strength, and excellent biocompatibility [11]. Other reports have revealed that HAp may induce the differentiation of dental pulp tissue into hard tissue-forming cells [12]. Additionally, HAp hastens the formation, precipitation, and deposition of calcium phosphate from bodily fluids [13], leading to the sealing of pulp against outside stimuli. However, HAp is highly brittle.

Previously, we developed biodegradable viscoelastic polymer materials for tissue-engineered medical devices with high affinity to fibroblasts [14]. The ductile properties of polymer contents help to overcome the poor fracture toughness of HAp. Combining HAp with our polymer may yield an effective carrier for capping materials. In this study, by changing the ratio of P(CL-*co*-DLLA) and HAp in the biodegradable material, we developed a carrier for pulp capping material. Further, as a combination of P(CL-*co*-DLLA) and HAp has not been reported, we evaluated the toxicity of P(CL-*co*-DLLA) and HAp to dental pulp cells and its effects on cell proliferation. BMP-2 has been previously reported to induce the differentiation of dental pulp tissue into hard tissue-forming cells, as well as tertiary dentin formation in vivo [15,16]. Therefore, a composite containing P(CL-*co*-DLLA) and HAp with BMP-2 was evaluated for direct pulp capping in a rat direct pulp capping model. Furthermore, we evaluated how the composite containing P(CL-*co*-DLLA) and HAp with BMP-2 acts on dental pulp cells by performing whole gene analysis using RNA sequencing.

The null hypothesis tested was that the novel polymer carrier is not suitable for direct pulp capping and does not support the induction of tertiary dentin after pulp exposure in rat teeth.

## 2. Materials and Methods

### 2.1. Ethical statement

The animal experimental protocol in this study was approved by the Ethical Guidelines Committee for Animal Care of Osaka University Graduate School of Dentistry (R-01-011-0). All surgeries were performed under general anesthesia, and all efforts were carried out to minimize pain or discomfort.

### 2.2. Cell Culture

We purchased human dental pulp stem cells (hDPSCs) collected from human sound third molars (Lonza, Basel, Switzerland). These cells have been reported to contain mesenchymal-like cells. Surface antigens on these cells have been confirmed for cluster designation (CD) 29+, CD73+, CD90+, CD105+, CD166+, CD34-, CD45-, and CD133- by flow cytometry. Cells were collected (TrypLE Select, Life Technologies, Carlsbad, CA, USA) when they reached 80% confluence. For the following assay, hDPSCs (passages 2–4) were cultured in α-Minimum Essential Media (α-MEM; Life Technologies, Carlsbad, CA, USA) supplemented with penicillin-streptomycin 10 µg/mL (Sigma-Aldrich, St. Louis, MO, USA). In the in vitro assays, 1% fetal bovine serum (FBS; Sigma-Aldrich, St. Louis, MO, USA) was used.

### 2.3. Material Preparation and Releasing Assay

A four-branched copolymer, ploy (ε-caprolactone-*co*-d,l-lactide) (P(CL-*co*-DLLA)) (CL:DLLA = 60:40), was synthesized by ring-opening copolymerization according to a previously described method [14,17,18]. The basic structure was evaluated by ^1^H NMR (400 MHz) (Bruker Nano GmbH, Berlin, Germany). The molecular weight and its distribution was estimated by gel permeation chromatography (GPC; Hitachi High-Tech Corporation, Tokyo, Japan) using a THF mobile phase at 40 °C through a combination of GL-R440, GL-R450, and GL-R400M Gelpack columns (GL science, Tokyo, Japan) at 2.05 mL/min. The relative molar mass was determined from a calibration curve created using the PS standards.

Chloroform-d solvent was used as received. To create novel polymer carrier for direct pulp capping (NPC), P(CL-*co*-DLLA) and hydroxyapatite (18736-14) (100-35-mesh, average particle size 4-6 µm) (Nacalai Tesque, Kyoto, Japan) were mixed with the following different ratios (P(CL-*co*-DLLA):hydroxyapatite) 50:50, 45:55, 40:60, 35:65, 30:70, and 20:80. Each material was then placed in a polytetrafluoroethylene mold (diameter of 3 mm and thickness of 1 mm). Material discs were incubated at 37 °C in a humidified chamber for 24 h. Fresh discs were sterilized with ethylene oxide gas before being inserted into the cell culture plates.

For the in vivo tests, BMP-2 (HZ-1128) (Cosmo Bio) was mixed with the NPC (50:50) to a concentration of 0.5 µg/mg manually in sterilized conditions. 

### 2.4. Lactate Dehydrogenase (LDH) Cell Cytotoxicity Assay

The biocompatibility of NPC to hDPSCs was evaluated using the LDH cytotoxicity assay (Thermo Fisher Scientific, Waltham, MA, USA) in accordance with the manufacturer’s protocol. Briefly, 3.0 × 10^6^ hDPSCs were seeded in a 35 mm dish containing a cell culture medium with an NPC disc of any ratio. A group without material disc was used as the control. Following incubation time points (1, 3, and 7 days), the supernatant was collected from each dish and centrifuged at 250 g for 5 min. The supernatant was then added to an LDH agent and incubated for 30 min away from light. The absorbance of the LDH reaction was measured at 405 nm using a microplate reader (ARVO MX, PerkinElmer, Waltham, MA, USA). Eight wells were measured for each supernatant group. Experiments were performed in triplicate.

### 2.5. Proliferation Assay and Cell Morphology

To determine the effects of NPC on cell growth, cell number counting was measured. Seeded hDPSCs (1.0 × 10^5^ cells) in medium supplemented with 1% FBS with each disk were evaluated by trypan blue exclusion at days 3 and 5. Cell morphology following co-culture with each disk was observed using a light microscope (ECLIPSE CI-L, Nikon, Tokyo, Japan) after 5 days of culture. Experiments were performed in triplicate.

### 2.6. Microstructure Analysis Using Scanning Electron Microscopy (SEM) 

Cultured cells that were incubated with composite P(CL-co-DLLA)) and hydroxyapatite were fixed using 4% paraformaldehyde before drying. After co-cultured with hDPSCs and NPC (ratio = 50:50) for 7 days, they were observed by backscattered electron diffraction (BSED) with SEM TM3000 (Hitachi High-Tech Corporation, Tokyo, Japan) using an accelerating voltage set at 15 keV.

### 2.7. Pulp Capping Procedure

Twenty Wistar rats (weighing 190–220 g; CLEA Japan, Inc., Tokyo, Japan) were used for in vivo experiments. Forty maxillary first molars were randomly divided into five groups: NPC 50:50, 45:55, 40:60, and 50:50 with BMP-2 and ProRoot MTA in direct pulp capping to evaluate mineralized tissue formation using each pulp capping material at 4 weeks (*n* = 8 for each group). 

Thereafter, the pulp capping procedure was performed as previously described [19]. Briefly, after anesthetizing, experimental teeth were cleaned and placed on a rubber dam sheet (Heraeus Kulzer, South Bend, IN, USA) with a custom-made rubber dam clamp (YDM, Tokyo, Japan) to perform sterile treatment. A Class I cavity was prepared on the occlusal surface with pulp exposure using a steel #1 round bur (Dentsply Sirona, Ballaigues, Switzerland) under sterile saline spray by an experienced operator to establish a stable and standard-sized cavity. Subsequently, the exposed pulp was directly capped with several NPCs, NPC (50:50) with BMP-2 or MTA (WMTA; Dentsply Sirona, OK, Lot 108824) using and following the manufacturer’s protocol. Next, the cavity was filled with glass ionomer cement (Fuji IX; GC International Corp, Tokyo, Japan) as temporary sealing. the opposing mandibular tooth were extracted by an adjusted house plier (Shoufu, Kyoto, Japan) to minimize occlusal forces.

### 2.8. Three-Dimensional Micro-Computed Tomographic (Micro-CT) Analyses

The animals were sacrificed at 4 weeks after direct pulp capping. The induced tertiary dentin was measured using a micro-CT scanner (R_mCT2; Rigaku, Tokyo, Japan) with a scanning resolution of about 10 μm intervals in an individual image as previously described [20]. The basic parameters of the scanner were as follows: voltage 90 kV, current 160 µA, and exposure time about 3 min in drying conditions. After scanning, 512 consecutive tomographic slice images were obtained. Next, image data were reconstructed using three-dimensional reconstruction imaging software (TRI/3D-BON; Ratoc System Engineering, Tokyo, Japan). Furthermore, the region of interest (ROI) for structural morphometry is defined from the mesial pulp horn in sagittal images. Each ROI was analyzed according to tertiary dentin volume (DV) at 4 weeks.

### 2.9. Histological Evaluation

Samples were stained with hematoxylin and eosin (HE) to confirm micro-CT results. Maxillary molars were dissected and fixed in 4% paraformaldehyde for 24 h at 4 °C. Experimental teeth were demineralized in Kalkitox solution (Wako, Osaka, Japan) following the manufacturer’s protocol for 1 day at 4 °C and then embedded in paraffin. Sagittal sections (10 μm thick) were stained with HE. Formation of tertiary dentine was quantified as previously described [21]. The criteria used for histological evaluation are shown in Table 1.

### 2.10. RNA Extraction and Quantitative Real-Time Polymerase Chain Reaction

The hDPSCs were seeded in 35 mm cell culture plates at a density of 30 × 10^6^ cells and in 1% FBS media with an NPC with BMP-2 and NPC specimen in the culture media for 7 days. Untreated hDPSCs were used as a control. Total RNA was prepared using the RNeasy Mini RNA isolation kit (Qiagen, N.V, Hilden, Germany) for RNA sequence analysis. RNA integrity was assessed with a 2100 bioanalyzer (Agilent Technologies, Santa Clara, CA, USA). Directional RNA-Seq libraries were created using the TruSeq stranded mRNA sample PrepKit (IlluminaInc., SanDiego, CA, USA), in accordance with the manufacturer’s recommendations. Library sequencing was carried out using the Illumina HiSeq 2500 system (IlluminaInc., SanDiego, CA, USA) with 75 bp single-end reads. Raw reads were deposited into the DNA Date Bank of Japan (DDBJ) sequence read archive (DRA, accession number: DRA009898). Data generated in the standard SangerFastQ format and phred-type quality scores Q30 were used for quality trimming. RNA-Seq reads were mapped to the GRCh38/hg38 human genome assembly, using the commercially available CLC Genomics Workbench (version9.5.2, CLCbio, Aarhus, Denmark). Read counts and fragments per kilobase of gene per million read values were used for heatmap visualization with integrated differential expression and pathway analysis (iDEP, PMID:30567491) and ClustVis (PMID:25969447), respectively.

### 2.11. Statistical Analysis

Significant differences were evaluated by one-way ANOVA with Tukey’s post-hoc test, the Kruskal–Wallis test, or the Steel-Dwass test. *p* values < 0.05 were considered statistically significant.

## 3. Results

### 3.1. General Properties of Polymer Materials

**Figure 1:**
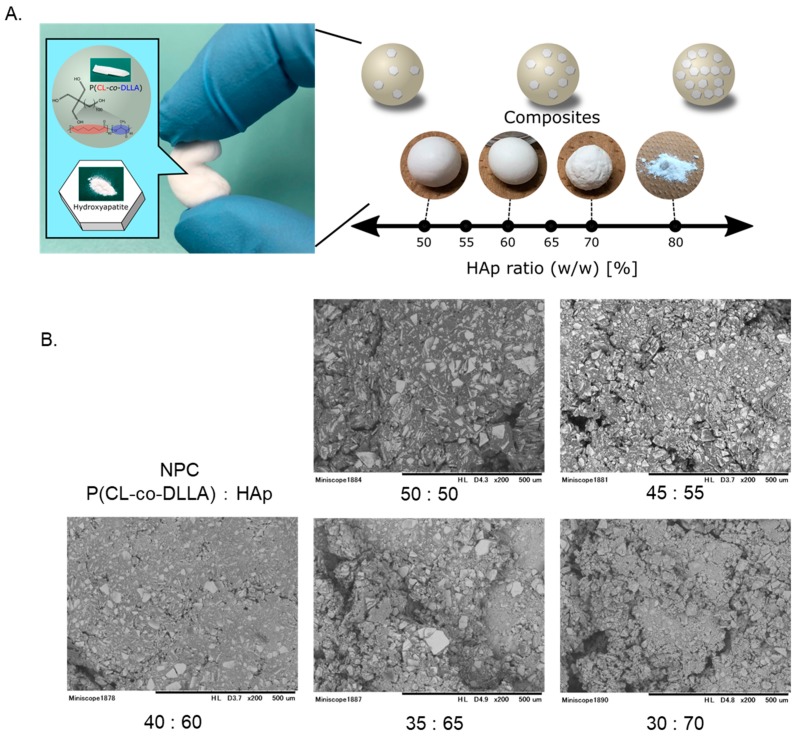
Fabrication of novel polymer carrier for pulp capping. Representative image of each HAp ratio (**A**). HAp ratios of 50% to 60% were good for application handling. Representative SEM images (200×) are shown (**B**). An HAp ratio higher than 65% could not be maintained. HAp= hydroxyapatite, NPC= novel polymer carrier for pulp capping, CL = ε-caprolactone, DLLA = d,l-lactide.

We aimed for a material form with high operability by changing the amounts of polymer and Hap (Figure 1). The polymer’s characteristics are listed in Supplemental Figure S1 and Table S1. When the ratio of HAp is 65% or more, it is difficult to maintain the original form, easily leading to collapse. When the ratio of HAp is 50% or less, it is easy to maintain the form, but the stickiness increases. This means that operation with the instrument is difficult. Therefore, NPCs of the ratios 50:50, 45:55, and 40: 60 were evaluated using the following assay.

### 3.2. Microstructural Analysis Using SEM

**Figure 2:**
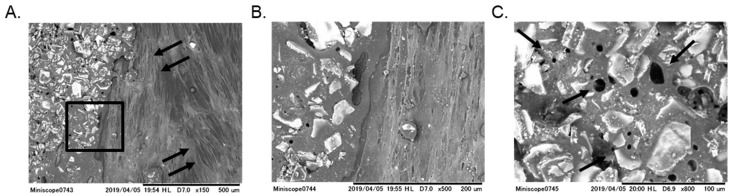
Co-culturing with human dental pulp stem cells (hDPSCs). Representative image of hDPSCs with NPC (50:50) for 7 days (**A**–**C**).

Representative SEM images of NPC before cell culturing are shown in Figure 2. The even NPC (50:50) consisted mostly of HAp. This trend did not change as the proportion of HAp increased. After co-culturing with hDPSCs, NPC was confirmed to be partially biodegradable (Figure 2A–C). A circular defect is considered as a biodegraded part (C, Black arrow). Cells growing in close contact with the NPC were not atrophied and showed a spindle shape (A, black arrows).

### 3.3. LDH Cytotoxicity and Proliferation Assay

**Figure 3:**
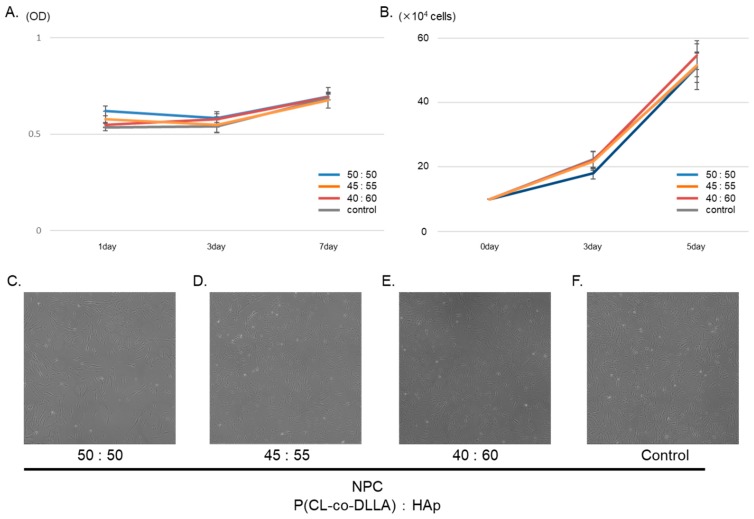
Effect of NPC on hDPSC biocompatibility in vitro. The LDH assay was used to evaluate cell cytotoxicity (**A**). NPC (50:50) tended to be more cytotoxic than other groups after one day. After the first day, it showed the same tendency as other groups. Cell counting was performed to evaluate cell proliferation (**B**). All groups showed similar behavior for cell proliferation after 3 and 5 days. Cell morphology was similar between all groups (**C**–**F**).

The cytotoxicity of each NPC disk was compared to the control group up to the 7th day. One NPC (ratio 50:50) was more cytotoxic on the first day compared to the other groups (Figure 3A).

Then, to evaluate proliferation of hDPSCs, a cell counting assay was performed for 3 days and 5 days. hDPSC proliferation in the NPC co-culture group showed similar behavior compared to control (Figure 3B). Under observation, cultured hDPSCs with NPC were spindle shaped, and no differences in morphology or floating cells were observed compared to the control (Figure 3C–F). According to the results of biocompatibility assays, we concluded that NPC could be applied as a carrier for direct pulp capping. 

### 3.4. Three-Dimensional Micro-CT Analysis of Tertiary Dentin Formation

**Figure 4:**
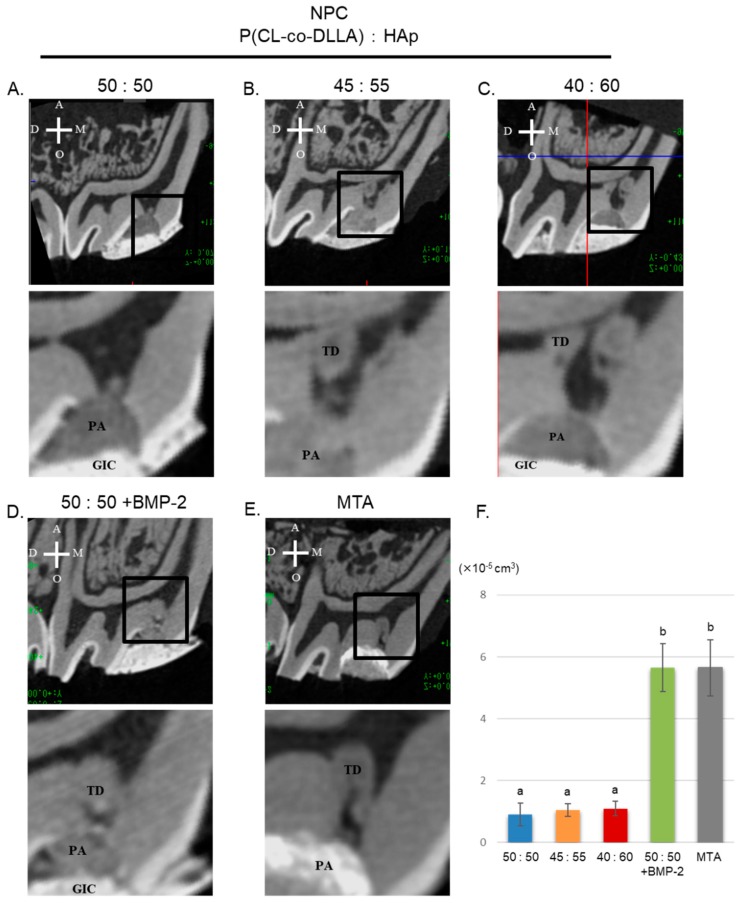
Micro-CT results after direct pulp capping in 4-week samples. Representative sagittal micro-CT images of tertiary dentin induced by several ratios of HAp NPC, NPC with BMP-2, and MTA are shown (**A**–**E**). The area surrounded by the black box is enlarged and shown at the bottom respectively). Tertiary dentin formation induced by NPC (50:50) with BMP-2 and MTA showed similar characteristics in dentin volume (DV) (**F**). A = apical, O = occlusal, M = mesial, D = distal, TD= Tertiary dentin, GIC = glass ionomer cements, PA = Pulp capping agent, P = pulp.

Representative micro-CT images of NPC, NPC (ratio 50:50) with BMP-2, and MTA are shown in Figure 4A–E. NPC with BMP-2 and MTA showed similar abilities in inducing tertiary dentin volume (DV) in exposed pulp tissue (F). NPC could not induce tertiary dentin in the exposed pulp area at 4 weeks. 

### 3.5. Histological Evaluation of Tertiary Dentin Formation 

**Figure 5:**
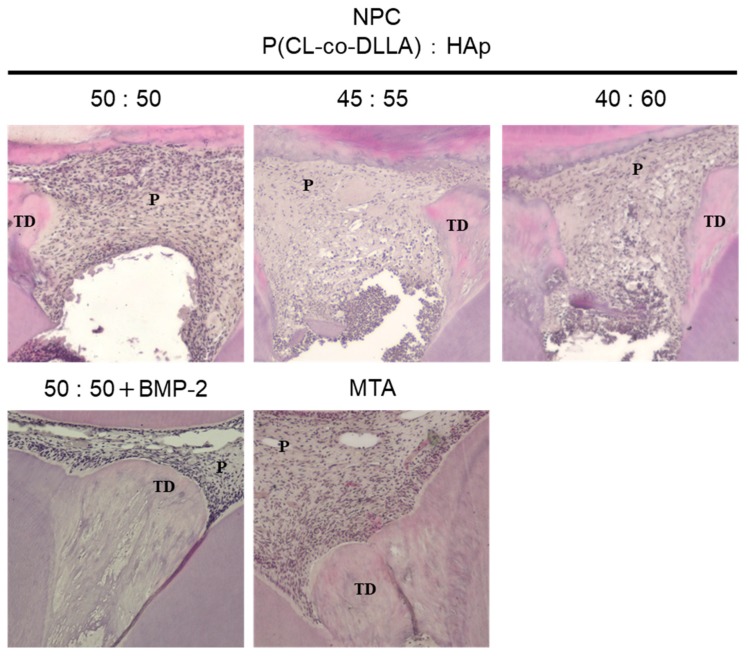
Representative histological images (HE staining) of tertiary dentine. Images taken after capping with several ratios of HAp NPC, NPC (50:50) with BMP-2, and MTA for 4 weeks. Sagittal sections are shown. The tertiary dentine covered application area was induced beneath NPC (50:50) with BMP-2 and MTA. A, apical; C, cavity; D, distal; M, mesial; O, occlusal; P, pulp; TD, tertiary dentine.

NPC with BMP-2 and MTA induced tertiary dentine formation with tubular structures beneath the direct pulp capping agent in pulp tissue (Figure 5). However, NPC (50:50, 45:55, and 40:60) mainly failed to induce tertiary dentine formation. Based on histological criteria, no significant differences were observed in the quality of tertiary dentine induced by NPC with BMP-2 and MTA after 4 weeks (Table 2). Pulp inflammation was not evaluated in this study at 4 weeks, as severe pulpal inflammation such as abscess formation was not observed.

### 3.6. RNA Sequence Analysis

**Figure 6:**
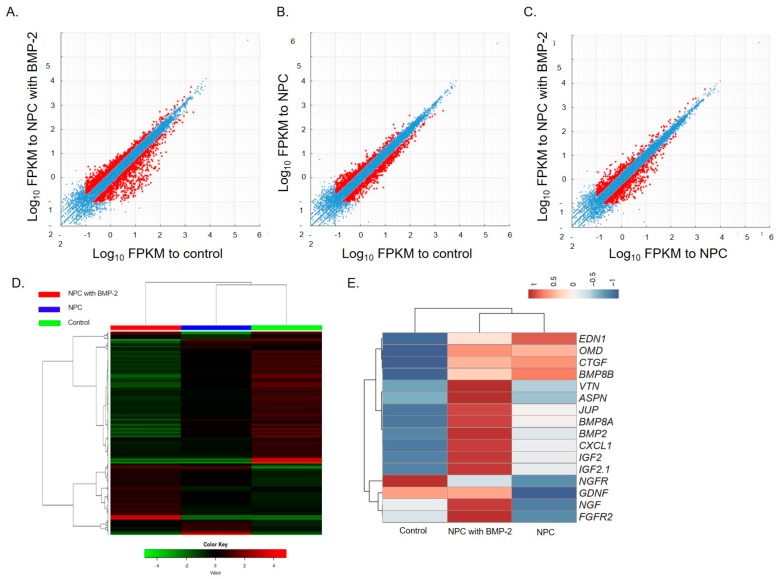
RNA-Sequence global reports. Scatter plots comparing gene expression patterns between NPC with BMP-2, NPC, and control (**A**–**C**). Heat map showing clustering of the 1000 most variable genes expressed in all samples (**D**). Each column represents one sample, and each row represents one gene. Hierarchical clustering was illustrated using the average linkage method with correlation distance. Color coding is based on empirical analysis of digital gene expression data in R (edgeR) log-transformed read count values. The color key indicates Z-scores that display the relative values of all tiles within all samples; green indicates lowest expression, black indicates intermediate expression, and red indicates highest expression. Heat map of key transcripts related to the osteo/dentinogenic differentiation process (**E**). Fragments per kilobase of gene per million reads (FPKM) values were used for heatmap visualization. FPKM values were collected from genes involved in the osteo/dentinogenic differentiation process. The heatmap was visualized by use of the web tool ClustVis (http://biit.cs.ut.ee/clustvis/) with default parameters. Red and blue indicate induced and repressed genes, respectively.

The effect of NPC with BMP-2 and NPC on hDPSC global gene expression was evaluated by RNA sequencing to determine whether NPC can act as a carrier. Scatter plots comparing global gene expression patterns between NPC with BMP-2 and the control, and between NPC and the control, are shown in Figure 6A–C. Red dots indicate genes’ up or downregulation by at least 2-fold in each dataset. The heat map in Figure 6D shows the clustering of 1000 of the most variable genes expressed in all samples. Each column represents a sample, and each row represents a gene. These results showed that NPC with BMP-2 regulated gene expression in hDPSCs but had different patterns of regulation compared to NPC. A heat map of the key transcripts for the osteo/dentinogenic differentiation process is shown in Figure 6E. These genes were consistent with those related to osteo/dentinogenic differentiation that were identified in previous reports [22,23,24,25,26,27,28,29,30,31,32,33].

## 4. Discussion

The mechanism of wound healing in the pulp has not yet been elucidated. Therefore, it is not clear whether MTA, currently used as the gold standard, is the best material for pulp wound healing. Therefore, development of a pulp capping material based on the wound healing mechanism of the pulp may dramatically improve the prognosis of pulp capping. Recently, there have been studies to examine the reaction of dental pulp tissue by combining various growth factors and molecules with materials [34]. This is valuable not only for the development of pulp capping materials, but also for clarifying the mechanism of early pulp healing. However, materials combined most often are gels and pastes, and although they have excellent growth factor release performance, they are not intended for clinical applications in dentistry, due to concerns about their mechanical strength.

Examination of several combinations of polymer and HAp showed that NPCs with less than 50% HAp were sticky and performed poorly as dental material. With an HAp ratio of 65% or more, HA brittleness is notable, and it plays only a minor role as a carrier (Figure 1A). Therefore, NPCs with HAp ratios of 50%, 55%, and 60% were determined, which would possibly function as direct pulp capping carriers. From SEM observation, the appearance was mostly influenced by HAp, even at a volume ratio of 50:50. It seemed that a polymer worked like an adhesive (Figure 1B). This fact explains the inability of the HAp ratio of 65% or more to maintain its form.

Next, we examined whether NPCs are useful as carriers for biological molecule release. Release assays were performed using NPCs (50:50) that mixed with fluorophores of different molecular weights, such as fluorescein (Mw: 332.31), fluorescein-labeled peptide (Mw: 993.03), and fluorescein-labeled albumin (Mw: 66,000) without cell culturing. The results showed that the release did not depend on the molecular weight, and that the fluorescein-labeled peptide released the slowest (Supplemental Figure S2). When comparing fluorescein-labeled albumin to fluorescein-labeled peptides, fluorescein-labeled albumin was released faster. This could be due to the fact that fluorescein-labeled albumin has a higher molecular weight and thus has a lower dispersibility in NPC, and therefore, we assumed that fluorescein-labeled albumin was released immediately during the degradation of the NPCs. When comparing fluorescein to fluorescein-labeled peptides, the release of fluorescein was faster, although it was three times lighter. This could be due to the amino acid properties of the peptide. When this material was co-cultured with cells, dissolution of the biodegradable polymer was observed, suggesting that the incorporated molecules would be released due to the dissolution (Figure 2A–C). As the releasing performances of the biological molecules may differ according to the amino acid sequences and conditions applied, it is necessary to validate the release test for each biological molecule in detail for future work.

Next, cytotoxicity and proliferation assays were performed to evaluate the biocompatibility of NPC with hDPSCs. NPC (50:50) tended to be more cytotoxic on day 1. Initial cytotoxicity may be due to the degradation of the biodegradable contents. Its negative effect also affected cell proliferation, but there was no statistical difference, and it was considered that long-term culture had no effect. Initial cytotoxicity may be due to degradation of the biodegradable contents.

These results indicate that NPC may be an effective carrier for the pulp capping material using growth factors or biological molecules. Therefore, whether this material was effective as a carrier releasing BMP-2 exhibiting hard tissue-inducing ability was evaluated in an animal model. NPC (50:50) was combined with BMP-2 and evaluated in a rat model because of the handling convenience among NPCs (50:50, 45:55, and 40:60).

**Figure 7:**
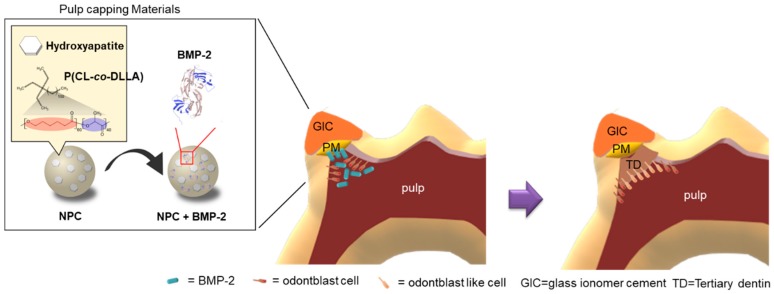
Proposed model for the NPC with BMP-2 effect on the wound healing of pulp tissue. After direct pulp capping, the high biocompatibility of NPC does not induce an abnormal inflammatory response. BMP-2 released from NPC is biodegradable and diffuses into the pulp tissue. Pulp cells upregulate the expression of genes related to the osteo/dentinogenic differentiation process and may also contribute to activate wound healing. As a result of these processes, mineralized tissue is induced beneath NPC with BMP-2.

NPC with BMP-2-induced tertiary dentin at a frequency similar to that of MTA (Table 2), and the amount of formation was similar to that of MTA in rat models (Figure 4). These results are supported by histological studies (Figure 5). Additionally, no severe inflammatory response or abscess was observed in the MTA group, or in the NPC group. This demonstrates that the NPC has a high biocompatibility. RNA sequencing also showed that compared to NPC, NPC with BMP-2 affected the pulp gene expression (Figure 6). NPC with BMP-2 increases the expression of many genes related to hard tissue formation of hDPSCs, compared with the other group (Figure 6E). These results may be attributed to the effect of BMP released from the carrier, leading to hard tissue induction in animal experiments. To date, there are few reports regarding whole gene expression in pulp cells with direct pulp capping material interaction. Raw reads data were deposited into the DDBJ sequence read archive (DRA, accession number: DRA009898). Accumulation of such a meta dataset could contribute to a novel direct pulp capping development in the future. In the present study, BMP-2, reported to induce hard tissue, was applied to evaluate the effectiveness of NPC as a pulp capping carrier. Based on our findings, the combination of NPC and BMP-2 may activate to the wound healing process within the pulp tissue (Figure 7). By combining this polymer with various growth factors or biological molecules, we can contribute to pulp research by studying the pulp’s response. On the other hand, since growth factors are generally effective and are unstable, they are less expensive peptides that are potential candidates to be combined with this material. Peptides with hard tissue-inducing ability can also be introduced [35,36]; further study is necessary because combinations with these peptides may be effective for novel pulp capping material in the future.

## 5. Conclusions

We made a novel polymer carrier, P(CL-*co-*DLLA)-HAp composite with BMP-2-induced tertiary dentin in rat molars, similar to MTA. RNA sequencing demonstrated that NPC worked as a carrier for direct pulp capping. Thus, the null hypothesis showed that the novel polymer carrier is not suitable for direct pulp capping, and it did not support the induction of tertiary dentin after pulp exposure was rejected.

## Figures and Tables

**Table 1:** The criteria used for the histological analysis of tertiary dentin formation.

Grade	Tertiary Dentin Formation
2	Consecutive hard tissue deposition without cellular contents or tunnel-like defect
1	Inconsecutive hard tissue deposition with cellular contents or tunnel-like defect
0	No hard tissue deposition

**Table 2:** Summary of the histological score for tertiary dentin formation.

Group Samples		Samples	Teritiary Dentin		
4 weeks			0	1	2
NPC (50:50)	a	8	5	3	0
NPC (55:45)	a	8	4	4	0
NPC (60:40)	a	8	4	4	0
NPC (50:50) with BMP-2	b	8	0	1	7
MTA	b	8	0	1	7

The criteria for scoring are shown in Supplemental Table S1. Groups with the same lowercase letters (i.e., a and b) are not significantly different.

## Supplementary Materials

The following are available online at https://www.mdpi.com/2073-4360/12/4/937/s1, Figure S1: 1H NMR spectrum of P(CL-co-DLLA); Figure S2. Performance of releasing assay without cell condition. In vitro release kinetics (n = 3) from the different composites, shown in percent molecule cumulative release; Table S1: Properties of P(CL-co-DLLA).
