# Supplementary material for: Performance of a Biodegradable Composite with Hydroxyapatite as a Scaffold in Pulp Tissue Repair
Source: Polymers (Basel). 2020 Apr 17;12(4):937. doi: 10.3390/polym12040937 (PMC7240495; doi:10.3390/polym12040937)
Supplement: Supplementary file 1 [file polymers-12-00937-s001.pdf]

polymers

MDPI

*Article*

# Performance of A Biodegradable Composite with Hydroxyapatite as A Scaffold in Pulp Tissue Repair

**Motoki Okamoto [1,*], Sayako Matsumoto [1], Ayato Sugiyama [2], Kei Kanie [2], Masakatsu Watanabe [1], Hailing Huang [1], Manahil Ali [1], Yuki Ito [1], Jiro Miura [3], Yujiro Hirose [4], Koichiro Uto [5], Mitsuhiro Ebara [5], Ryuji Kato [2,6], Aika Yamawaki-Ogata [7], Yuji Narita, Shigetada Kawabata [4], Yusuke Takahashi [1] and Mikako Hayashi [1]**

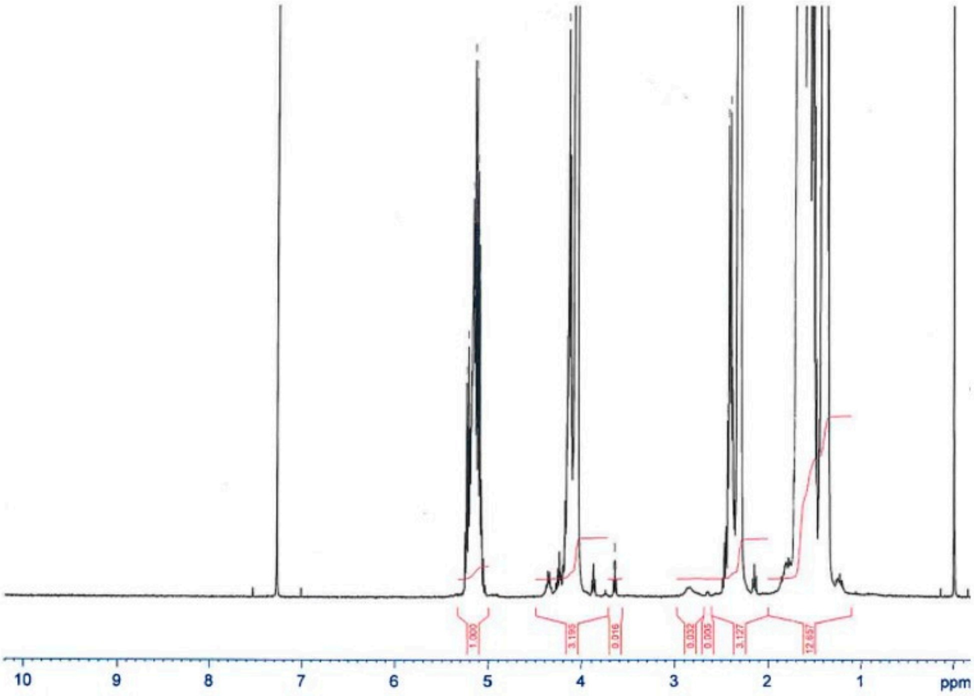


**Figure S1.** 1H NMR spectrum of P(CL-co-DLLA).

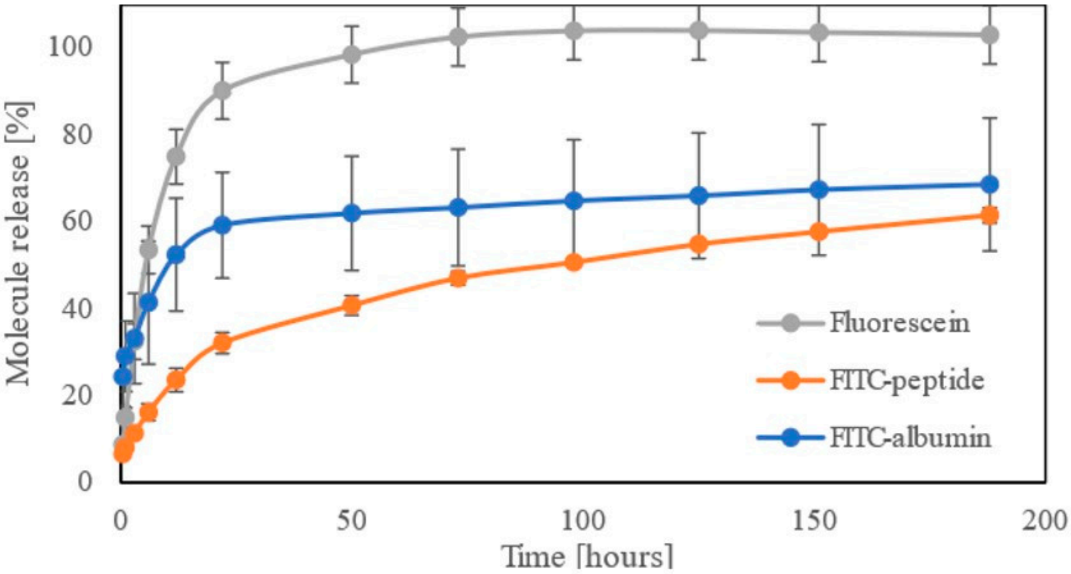


**Figure S2.** Performance of releasing assay without cell condition. In vitro release kinetics ($n = 3$) from the different composites, shown in percent molecule cumulative release. Materials and methods are

shown below. For the releasing assay, the in vitro molecular release was analyzed by missing various fluorescent-labeled molecules and observing the fluorescence intensity for one week. First, Fluorescein (46955, Sigma-Aldrich, Molecular weight: 332.31), fluorescent-labeled peptide (FITC-Ahx-RGDSG-OH, GL Biochem Ltd., shanghai, Molecular weight: 993.03), and fluorescent-labeled albumin (A9771, Sigma-Aldrich, molecular weight: 66000) were mixed with the composite (P(CL-co-DLLA):hydroxyapatite = 50:50) at 2.18, 2.18 μmol/g, and 0.5 mg/g respectively. Samples used for the experiment were prepared in a disc shape with a diameter of 6 mm and a thickness of 300 μm and were exposed to 100 μL of PBS (14249-95, Nacalai Tesque) at 37 °C. At each time point of measurement, the exposed PBS was mixed with 50 μL of 0.1 M NaOH, THE FLUORESCENCE value was measured using a plate reader (Fluoroskan Ascent L5210470, Labsystems) (Ex/Em:485/538), and samples were soaked in new PBS. The amount of molecular release was calculated from the measured fluorescent value using each standard curve. The ratio of molecular release (%) was calculated by dividing the amount of molecular release by the amount of total molecules in the initial composite.

**Table S1.** Properties of P(CL-co-DLLA).

| **In copolymer** | | | **Copolymer** | | | |
|---|---|---|---|---|---|---|
| CL/DLLA | CL (mol%) [a] | DLA (mol%) [a] | Mt [b] | Mw [c] | Mn [c] | Mw/Mn [c] |
| 60/40 | 61 | 39 | 39000 | 29900 | 19500 | 1.53 |

[a] Determined by 1H NMR (solvent: CDCl3); [b] Theoretical molecular weight; [c] Estimated by GPC (solvent: THF, standard: Polystyrene).
